# Editorial: Microbe-environment interaction across grassland ecosystems: soil microbial diversity, plant-microbe interplay, and ecosystem functionality

**DOI:** 10.3389/fmicb.2026.1971028

**Published:** 2026-09-28

**Authors:** Kamran Malik, Anum Ahmad, Muhammad Usman

**Affiliations:** 1 State Key Laboratory of Herbage Improvement and Grassland Agro-ecosystems, Lanzhou, China; 2 Key Laboratory of Grassland Livestock Industry Innovation, Ministry of Agriculture and Rural Affairs, Lanzhou, China; 3 Engineering Research Center of Grassland Industry, Ministry of Education, Lanzhou, China; 4 Gansu Tech Innovation Centre of Western China Grassland Industry, Lanzhou, China; 5 Centre for Grassland Microbiome, Lanzhou, China; 6 College of Pastoral Agriculture Science and Technology, Lanzhou University, Lanzhou, China; 7 The Roslin Institute, University of Edinburgh, Easter Bush Campus, Midlothian, United Kingdom; 8 Guangdong Provincial Key Laboratory of Marine Biotechnology, Shantou University, Shantou, China

**Keywords:** grassland ecosystem, grass–microbe interactions, microbial community diversity, nutrient cycling, plant growth

## Introduction

The grassland ecosystem is shaped by continuous interactions between the grassland plants, soil microorganisms, grazing, and their surrounding environments. However, the mechanisms through which microbial communities respond to environmental gradients, vegetation changes, management practices, and ecosystem disturbances remain incompletely understood. Understanding these microbe-environment interactions in grasslands is essential for revealing how microbial diversity, community assembly, and functional traits contribute to ecosystem stability, nutrient cycling, and ecological restoration. This Research Topic aims to provide new insights into the dynamics of grassland microbiomes and explore how environmental factors regulate microbial diversity, plant-microbe relationships, and ecosystem functionality across different grassland systems. Overall, the studies contributing to this RT demonstrated that microbial diversity is not only related to local conditions but also to complex interactions between the soil properties, vegetation, nutrient availability, and ecological processes that influence microbial assembly and function that span the various grassland ecosystems.

Cai et al. showed that the soil microbial diversity is altered along the gradient mainly by soil nutrient levels and enzyme activities. The results also indicated that the edaphic factors, organic matter and available phosphorus, were important environmental filters affecting the assembly of the microbial community, suggesting that local edaphic conditions can play an important role in shaping microbial responses to environmental heterogeneity.

The findings by Zhou et al. indicated that short-term rest-grazing facilitated the recovery of the microbial community and soil inorganic nitrogen levels, with ammonium nitrogen as the main factor controlling microbial community structure. Further, the beneficial effect of nitrogen fertilization was limited by a decrease in total microbial biomass and a change in microbial regulation in response to the availability of ammonium compared to microbial biomass carbon, suggesting that about 30 days of grazing exclusion without nitrogen fertilization might be a better approach for restoring degraded Carex tibetikobresia meadows.

Wang et al. showed that in alpine grasslands, microbial community structure and functional potential changed quickly after one rainfall pulse, with greater changes occurring in the rhizosphere than in bulk soil communities. They also found that their metagenomic analysis identified carbon metabolism, amino acid degradation, and chemotaxis pathways that were increased with the addition of moisture, indicating that the microbiomes in the rhizosphere are sensitive to the short-term fluctuations that occur in their environment.

In Zou et al., it was illustrated that the rhizosphere microbial community underwent changes in loquat–tea intercropping, which led to a decrease in the concentration of functional microorganisms like Bradyrhizobium and Trebonia, and an increase in microbial diversity. Based on metagenomic analysis, they found that plant diversification can increase the metabolic potential of the microorganisms and the relationship between the main groups of microorganisms and soil nutrient characteristics, suggesting intercropping as a means to improve soil health and ecosystem functions.

Pang et al. showed that planting *V. nigrum* in Inner Mongolian Mountain steppe ecosystems significantly restructured the soil microbial community, increasing nutrient availability and changing the fungal diversity as well as the microbial interaction network. They found that fungal rather than bacterial communities were more responsive to vegetation growth, underscoring the critical role of the below-ground microbial response in understanding changes in ecosystems mediated by vegetation invasion.

Zheng et al. have shown that *Poria cocos* develops a unique microbiome, characterized by a unique enrichment of microbial taxa that were associated with positive enrichment of the accumulation of pachymic acid. Microbiome-informed cultivation strategies are emerging, and their study suggests host-associated microbiomes can play a role in regulating the production of beneficial fungal metabolites.

Yi et al. showed that parent soil type greatly influences the effect of ecological restoration practices on microbial responses in karst ecosystems. The results indicated that biochar and mowing promoted microbial abundance and diversity, especially in red soils, but also highlighted the need to consider how management practices affect the outcome of the restoration and the inherent soil properties.

Using integrated genomic and phylogenetic approaches, Iza-Arteaga et al. discovered a novel mycoparasitic fungus, *Trichoderma tlahuicanensis*, present in a traditional Mexican milpa ecosystem. Their results show the potential of unexplored microbial diversity in the sustainable management of plant diseases through ecological and biotechnological approaches.

In a study of soil bacterial communities across the Mu Us Sandy Land, Shang et al. found that these communities were highly structured across geographical landscapes, indicating the role of spatial heterogeneity and local environmental filtering in structuring microbial communities. They found that soil moisture, nutrient availability, and other physicochemical properties were significant factors in bacterial diversity and composition, and that there were some bacterial taxa with high habitat specificity. The results highlight the value of spatial heterogeneity in the evaluation of microbial responses to desertification and ecological restoration for relatively similar sandy ecosystems, with the possibility of different community structures arising in response to local environmental conditions.

Shan et al. revealed that the establishment of artificial grasslands can substantially modify soil organic carbon fractions as well as the communities of microorganisms involved in carbon fixation. The cbbL-type carbon-sequestering microbes changed with the changes in the pool of particulate carbon and mineral-associated carbon, showing that the changes in vegetation type can affect soil carbon storage by both changes in the amount of carbon inputs from plant materials and changes in the microbial carbon-fixation process. Their results show the necessity to account for different functional groups of microorganisms when assessing the potential of carbon sequestration and ecology of artificially created grasslands.

In addition to the loss of vegetation and soil resources, Qiao et al. noted that degradation of grassland is also a significant disruption of the underlying microbiome. They found that excessive grazing, droughts, salinization, and nutrient enrichment can lead to decreased microbial diversity, disrupted microbial interaction networks, and decreased plant–microbe interactions and nutrient cycling functions. Importantly, the authors contended that the restoration effort should not merely be directed toward restoring plant cover, but also microbial functions and interactions. Their proposed microbiome-informed restoration framework offers an important conceptual foundation for integrating the microbiome into future grassland restoration and management practices.

Liu et al. showed that nitrogen enrichment in temperate desert grasslands can significantly reduce the diversity and stability of soil bacteria and that simulated grazing can mitigate this impact to a certain extent. Grazing affected vegetation and soil parameters in a way that buffered some of the effects of nutrient enrichment, while nitrogen addition changed the structure of the bacterial community and decreased the stability of some of the microbial properties. These results are relevant to the current trends of elevated nitrogen deposition and altered grazing patterns and highlight the importance of understanding the effect of changes in multiple environmental stressors on the trajectory of global change in grassland microbial communities.

Wang et al. demonstrated that short-term rainfall events can quickly shift microbial community and functional potential in alpine grasslands. The rhizosphere had a more pronounced response to rainfall, which affected microbial communities and enhanced key functional pathways that involved carbon metabolism, amino acid degradation, and bacterial chemotaxis, while bulk soil communities were less susceptible. The increase in specific microbial groups after precipitation also indicated a quick microbial shift to the newly available resources. This study shows the importance of moisture pulses on microbial functions and interaction networks in the rhizosphere in only a few hours, and thus the sensitivity of the microbiomes to more and more variable precipitation in climate change.

Zhao et al. showed that the stability of microbial communities in the restoration of alpine grasslands is closely related to the structure of microbial interaction networks, not just microbial diversity. They identified separate stability trends in bacterial and fungal communities and demonstrated that network connectivity, strength of interactions, and topological vulnerability were significant factors in microbial resilience. The results offer a valuable contribution to restoration ecology as they indicate that a diverse microbial community is not a prerequisite for a stable ecosystem, but that the ecosystem functions should also be restored through strong microbial interactions that are able to withstand environmental disturbances.

Mingjun et al. increased the diversity of actinomycetes known to be associated with rhizosphere soil by isolating two new species, *Streptomyces pallidus* sp. nov. and *Streptomyces qianjiangensis* sp. nov., from the rhizosphere soil of *Cyclosorus acuminatus*. These strains were identified as new bacterial taxa using a combination of morphological, physiological, phylogenetic, and genomic analyses. The study not only underscores the taxonomic diversity of microorganisms in the rhizosphere but also highlights the vastness of microbial life found in plant habitats and the need to pay attention to the rhizosphere as a source of microorganisms that may have significant ecological and biotechnological roles.

Peng et al. revealed that the vulnerability of soil organic carbon pools is not shared by all, but particulate organic carbon (POC) and mineral-associated organic carbon (MAOC) exhibited different vulnerability patterns in Inner Mongolian grasslands. They concluded that particulate carbon was especially vulnerable to degradation of vegetation and its associated changes in organic matter inputs, while mineral-associated carbon was comparatively more stable. The study is able to differentiate between the various soil carbon pools and not use total organic carbon as a single carbon pool, thus providing a clearer understanding of the effect of grassland degradation on belowground carbon storage and the need for assessments of the various carbon pools when examining the long-term effects of desertification.

## Conclusion

The studies in this Research Topic highlight microorganisms as key regulators of grassland ecosystem functioning and demonstrate their dynamic response to both natural and anthropogenic environmental changes. Across different grassland ecosystems, the microbial communities were influenced by several factors, including grazing pressure, restoration methods, fertilization, soil properties, soil moisture, soil transformation, and altitude. These studies also emphasize that microbial diversity is not sufficient to explain ecosystem stability, but microbial interactions, functional traits, and network structure are important factors that contribute to the stability of the ecosystem. The results highlight the importance of considering microbial aspects in grassland conservation, restoration, and management.

The contributions in this Research Topic reflect the complexity of the interacting microorganisms that influence the grassland ecosystem functions. These studies extend our understanding of microbial diversity, plant–microbe interactions, the role of environmental factors, and overall ecosystem processes and functionality, highlighting the importance of integrated approaches for grassland conservation, restoration, and management strategies. Further studies combining multi-omics, long-term ecological monitoring, and control manipulations in grassland ecosystems will provide much deeper insight into microbial interactions with the environment and help in developing sustainable management strategies for grassland ecosystems.

